# Reductive Debromination of Polybrominated Diphenyl Ethers - Microbes, Processes and Dehalogenases

**DOI:** 10.3389/fmicb.2018.01292

**Published:** 2018-06-19

**Authors:** Siyan Zhao, Matthew J. Rogers, Chang Ding, Jianzhong He

**Affiliations:** ^1^Department of Civil and Environmental Engineering, National University of Singapore, Singapore, Singapore; ^2^Isotope Biogeochemistry, Helmholtz Centre for Environmental Research – UFZ, Leipzig, Germany

**Keywords:** flame retardants, organohalides, polybrominated diphenyl ethers (PBDEs), reductive debromination, debromination pathways, reductive dehalogenase genes

## Abstract

Extensive utilization of polybrominated diphenyl ethers (PBDEs) as flame retardants since the 1960s in a variety of commercial products has resulted in ubiquitous environmental distribution of commercial PBDE mixtures. Dangers posed to biological populations became apparent after the discovery of elevated levels of PBDEs in biota, most notably in human breast milk and tissues. Environmental persistence of PBDEs results in significant transboundary displacement, threatening fragile ecosystems globally. Despite efforts to curtail usage of PBDEs, public concern remains about the effects of legacy PBDEs contamination and continued discharge of PBDEs in regions lacking restrictions on usage and manufacture. Among available technologies for remediation of PBDEs such as *ex-situ* soil washing, electrokinetic degradation, and biodegradation, this review focuses on bioremediation by microbes under anaerobic conditions. Bioremediation is generally preferred as it is less disruptive to contaminated ecosystems, is cost-effective, and can be implemented at sites that may be inaccessible to more traditional *ex-situ* methods. The aims of this review are to (1) summarize current knowledge of anaerobic microbes that debrominate PBDEs and their associated synergistic partnerships with non-dehalogenating microbes; (2) explore current understandings of the metabolic reductive debromination of PBDE congeners; (3) discuss recent discoveries on dehalogenase genes involved in debromination of PBDEs.

## Introduction

Polybrominated diphenyl ethers (PBDEs) have been widely used as flame retardant additives in a variety of manufactured products, from paints and plastics to textiles and televisions since 1960s. Deposition of anthropogenic PBDEs has subsequently been identified in air, soils, and water across the world (McGrath et al., 2017), even in remote areas including isolated mountaintop sediments, the Faroe Islands, and the Antarctic (Lindström, 1999; Gallego et al., 2007; Wild et al., 2015). In recent years, PBDEs concentrations as high as 10,000 ng/g soil have been detected at manufacturing and e-recycling sites (Alabi et al., 2012; Labunska et al., 2013; Li et al., 2015; Deng et al., 2016). Notably, PBDEs have the tendency to accumulate in biota (de Boer et al., 1998; Boon et al., 2002; Norstrom et al., 2002; Zhu and Hites, 2004) and have diverse toxicological effects including endocrine disruption, metabolic disorders in biological populations, as well as neurological and developmental disorders in human (Siddiqi et al., 2003; Windham et al., 2015; ATSDR, 2017). Growing concerns over risks to human and environmental health have resulted in hepta- through tetra-BDEs being listed as persistent organic pollutants (POPs) on the United Nations Stockholm Convention in 2009 (United Nations Environment Programme, 2009).

Though restrictions and bans on manufacture and usage of PBDEs have been in place for several years, these legislations have no effect on the release of PBDEs from existing products or from recycled materials containing PBDEs. Environmental deposition of PBDEs can occur via release during manufacture and use of consumer products, improper disposal, and recycling of PBDEs containing products, volatilization during incineration, and discharge from wastewater treatment facilities. PBDEs are highly lipophilic, have low water solubility and low vapor pressures. These physical characteristics are largely dictated by the number and arrangement of bromine atoms on the molecule, with highly brominated PBDEs being more lipophilic, less soluble and less volatile (Table 1). Hence, highly brominated PBDEs adsorb readily to soils and sediments, where they persist and act as reservoirs leaching into other compartments over time (O'Driscoll et al., 2016). PBDEs also tend to bind to the organic fraction of particulate matter, soils, vegetation, and sediments following environmental deposition. Thus, PBDEs have a tendency to be exchanged between the atmosphere and surface, allowing passive transport over long distances.

**Table 1:** Physical properties of PBDE mixtures.

**Property**	**Penta-BDE**	**Octa-BDE**	**Deca-BDE**
Molecular weight	Mixture	Mixture	959.22
Color	Clear, amber to pale yellow	Off-white	Off-white
Physical state	Highly viscous liquid	Powder	Powder
Melting point	−7 to −3°C (commercial)	85–89°C (commercial); 200°C (range, 167–257); 79–87°C; 170–220°C	290–306°C
Boiling point	>300°C (decomposition starts above 200°C)	Decomposes at >330°C (commercial)	Decomposes at >320, >400, and 425°C
Density (g/mL)	2.28 at 25°C; 2.25–2.28	2.76; 2.8 (commercial)	3.0; 3.25
**Solubility:**			
– Water	13.3 μg/L (commercial);	< 1 ppb at 25°C (commercial);	< 0.1 μg/L
	2.4 μg/L (pentabromodiphenyl ether component);	1.98 μg/L (heptabromodiphenyl ether component)	
	10.9 μg/L (tetrabromodiphenyl ether component)		
– Organic solvent(s)	10 g/kg methanol; miscible in toluene	Acetone (20 g/L); benzene (200 g/L); methanol (2 g/L)—all at 25°C	d-limonene (0.1823 g/100 g solvent); n-propanol (0.1823 g/100 g solvent)—all at 20°C *
**Partition coefficients:**			
– Log K_ow_	6.64–6.97; 6.57 (commercial)	6.29 (commercial)	6.265
– Log K_oc_	4.89–5.10	5.92–6.22	6.8
Vapor pressure	2.2 × 10^−7^−5.5 × 10^−7^ mm Hg at 25°C; 3.5 × 10^−7^ mm Hg (commercial)	9.0 × 10^−10^–1.7 × 10^−9^ mm Hg at 25°C; 4.9 × 10^−8^ mm Hg at 21°C (commercial)	3.2 × 10^−8^ mm Hg
Henry's Law constant (atm-m^3^/mole)	1.2 × 10^−5^; 1.2 × 10^−6^; 3.5 × 10^−6^	7.5 × 10^−8^; 2.6 × 10^−7^	1.62 × 10^−6^; 1.93 × 10^−8^; 1.2 × 10^−8^; 4.4 × 10^−8^

Adapted from ATSDR (2017); * From Chen et al. (2016).

**Figure 1:**
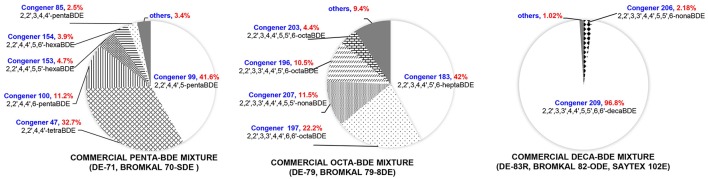
Compositions of representative penta-, octa-, and deca-BDE mixtures. Congeners below 2% (w/w) are considered as others. As a result of the chemical process used to generate PBDE mixtures, the exact congener composition remains undefined and may vary significantly between different manufacturers and production batches (La Guardia et al., 2006).

PBDE formulations have been primarily manufactured as three different technical mixtures (Figure 1): penta-BDEs, octa-BDEs, and deca-BDEs. Although BDE-209 has historically been the most widely used PBDE congener, BDE −47, −99, −100, and −153 are the most commonly observed PBDEs in the environment (Darnerud et al., 2001), indicating that environmental transformation of highly brominated BDEs is an important pathway contributing to the dispersal and environmental impact of PBDEs. The partial debromination of deca- and octa-BDEs is particularly worrisome because the less brominated metabolites like tetra- and penta-BDEs are of higher toxicity (Palm et al., 2002). Both abiotic and biotic processes are responsible for the breakdown of PBDEs in the environment. Photolysis, which can occur in the atmosphere and at soil surfaces, of lesser brominated BDEs (BDE-28, BDE-47, BDE-99, BDE-100, BDE-153, and BDE-183) (Rayne et al., 2006; Fang et al., 2008) and of BDE-209 (Stapleton and Dodder, 2008) has been demonstrated under laboratory conditions, although there is only weak evidence that atmospheric photodegradation is a major contributor to environmental attenuation of PBDEs (Schenker et al., 2008). Anaerobic and anoxic sediments and soils are major sinks and environmental reservoirs for PBDEs, making anoxic debromination by microorganisms an important route for eliminating PBDEs in the environment.

Debromination of a variety of PBDEs, including the three primary technical mixtures, has been observed in soil, sewage sludge, and estuarine and marine sediments under different environmental conditions (redox, pH, available electron donors, etc.), although the process is typically slow and often results in incomplete debromination of PBDEs. Despite these apparent limitations of anaerobic microbial degradation of PBDEs, bioremediation is considered the most environmentally friendly technology to remediate PBDEs. Although bioremediation requires extensive site characterization to be successful and is often much slower than traditional *ex-situ* treatment strategies, it is less expensive, less disruptive to sites, and can be more complete in degrading hazardous compounds. Considering the fragility and inaccessibility of many of the environments and ecosystems plagued by PBDEs contamination, bioremediation via anaerobic microbes is the best available option for eliminating PBDEs from contaminated areas.

This review aims to summarize current knowledge of microbial reductive debromination of PBDEs under anaerobic conditions, the debromination pathways involved, and dehalogenase genes identified so far. Investigation of PBDEs debrominating microbes and exploration of the underlying mechanisms of debromination will enable more effective tracking the fate of PBDEs in the environment.

## Microorganisms involved in reductive debromination of PBDEs

**Figure 2:**
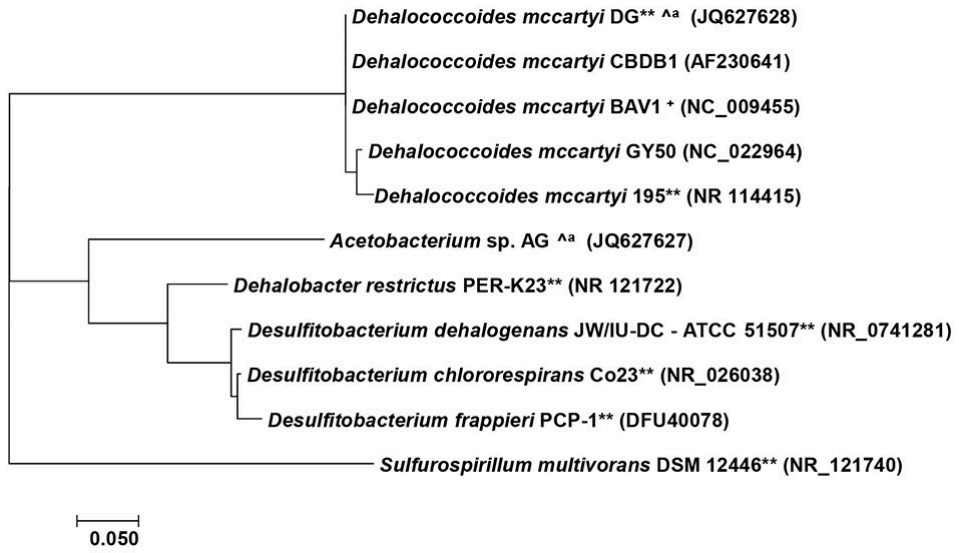
Phylogenetic analysis of PBDE debrominating bacteria. The tree was constructed with MEGA 7 (Kumar et al., 2016) using Neighbor-joining method in Kimura 2-parameter mode (Kimura, 1980). ^∧*a*^, derived from same culture, ^**^require auxiliary substrate, ^+^only in mixed culture.

The hydrophobicity of PBDEs impedes bioavailability, which results in low biomass of debrominating microorganisms. This low abundance is a critical challenge to enrichment, isolation, and characterization of PBDE debrominating bacteria. After pioneering efforts identified debromination of PBDEs in previously isolated organochlorine dehalogenating members of the genera *Sulfurospirillum* and *Dehalococcoides* (He et al., 2006) as well as *Desulfitobacterium* and *Dehalobacter* (Robrock et al., 2008), later studies successfully enriched and isolated PBDEs debrominating bacteria from multiple environmental sources (Lee et al., 2011; Ding et al., 2013, 2017) and confirmed the role of members of the genera *Dehalococcoides* and *Acetobacterium* in environmental debromination of PBDEs (Figure 2, Table 2).

**Table 2:** PBDE debrominating cultures.

	**Sample ID**	**Source**	**Auxiliary substrate**	**deca-BDEs**	**octa-BDEs**	**penta-BDEs**	**Remarks**	**Duration**	**Citation**
Pure cultures	*Dehalococcoides mccartyi* 195	Anaerobic sewage digestor sludge	TCE	No	hepta- through tetra-	N.A.		6 months	He et al., 2006
	*Dehalococcoides mccartyi* DG	Isolate from G	TCE	N.A.	penta- tetra-	tetra-	Minimal conversion; Lost octa-BDE debromination activity of parent culture, G, which can only be resumed by co-cultivating AG and DG;	Octa-BDEs 5 months; penta-BDEs 1 month	Ding et al., 2013
	*Acetobacterium* sp. AG	Isolate from G	Lactate	N.A.	penta- tetra-	penta- through di-			
	*Dehalococcoides mccartyi* CBDB1/n-ZVI	Saale river sediment	n-ZVI	20% conversion to diphenyl ether	N.A.	N.A.	No debromination of deca-BDE by CBDB1 without addition of n-ZVI; the role of CBDB1 in this study is uncertain due to pH and unbalanced hydrogen consumption.	1 month	Xu et al., 2014 Yang, 2017
	*Dehalococcoides mccartyi* GY50	Isolate from co-culture, GY2, sand, and silt near Lianjiang River	No	N.A.	N.A.	Diphenyl ether	GY2 share the same debromination activity with its isolate GY50; Complete debromination; *Ortho*-removal preferred; identified PBDE RDases, PbrA1, PbrA2, PbrA3	2 weeks	Lee et al., 2011; Ding et al., 2017
	*Dehalococcoides mccartyi* GY52	Variant of GY50 after consecutive transfer in TCE	No	N.A.	N.A.	Di-	A genome island where *pbrA1* and *pbrA2* locate was deleted from GY50	2 weeks	
	*Desulfitobacterium chlororespirans* Co23	2,3-CP dehalogenating compost soil	3-chloro-4-hyxdroxybenzoate	Similar debromination profile as ANAS195 (data not shown)				N.A.	Robrock et al., 2008
	*Desulfitobacterium halogenans* JW/IU-DC	Freshwater sediments-pond	3-chloro-4-hydroxphenylacetate	Similar debromination profile as ANAS195 (data not shown)				N.A.	Robrock et al., 2008
	*Desulfitobacteriun frappieri* PCP-1	A mixture of pentachlorophenol contaminated soil and anaerobic sewage sludge	pentachlorophenol	N.A.	hepta- hexa- penta-	penta 99 to tetra, tri, di; tetra 47 to tri and di	Debromination pathways were identified by spiking individual congeners; para- and meta-removal is preferred; debromination for higher brominated BDEs are slower;	3 months	Robrock et al., 2008
	*Dehalobacter restrictus* PER-K23	Anaerobic Rhine river sediment and ground anaerobic granular sludge	PCE	N.A.	hepta- hexa- penta-	penta 99 to tetra-; tetra47 to tri and di		3 months	Robrock et al., 2008
	*Sulfurospirillum multivorans* DSM12446	Activated sludge	TCE	Octa- Hepta-	No	N.A.		2 months	He et al., 2006
Mixed Cultures	Culture G	Soil samples from a river bank in Wisconsin	TCE	N.A.	hexa-, penta-, tetra-(dominant), tri-	penta- through di-	*Para*- and *meta*-removal preferred in octa-BDEs debromination; Penta-BDEs debromination similar as AG, strictly *para*-removal	octa-BDEs 5 months; penta-BDEs 1 month	Ding et al., 2013
	EC195	Highly enriched 195 containing autotrophic culture	TCE	No	hepta- hexa- penta- tetra- di-	N.A.	Additional one hexa and two penta, as well as tetra and di congeners produced compared with strain 195	3 months	He et al., 2006
	ANAS195	An enrichment culture with strain 195	TCE	No	hepta- through di-	N.A.	Faster and more extensive debromination compared with strain195	3 months	He et al., 2006
	EC195+BAV1	EC195 with strain BAV1	TCE	No	tetra- tri- di-	N.A.	BAV1 shows no debromination on deca- or octa-BDEs. More extensive debromination compared with EC195 suggests BAV1 can debrominate lesser brominated PBDEs	3 months	He et al., 2006
Microcosms	Plug-flow bioreactor with colonization water from wetland near munition dump		No	N.A.	N.A.	N.A.	Di-BDE 15 to mono-BDE 3 and diphenyl ether	12% and 61% conversion with HRT 3.4 h and 6.8 h, respectively	Rayne et al., 2003
	Sewage sludge from mesophilic digester in Dubendorf, Switzerland		4-bromobenzoic acid, 2,6-dibromobiphenyl, tetrabromobisphenyl A, hexabromocyclododecan, and decabromobiphenyl	Nona- Octa-	N.A.	N.A.	*Para*- and *meta*-removal preferred	238 days	Gerecke et al., 2005
	Loam sediment from Celey Bog Park, West Lafayette, IN, USA		Methanol and dextrose	Nona- through Hexa-	N.A.	Tetra- Tri-	*Para*-removal preferred	8 months for penta- and tetra-BDEs, 3.5 years for deca-BDE	Tokarz et al., 2008
	A few soils and sediments from multiple locations in China, Singapore, and US		No	N.A.	Hexa- Penta- Tetra-	N.A.	*Dehalococcoides* exist in 11 out of 14 active microcosms; more extensive debromination was observed with TCE as auxiliary substrate	2 months	Lee and He, 2010
			TCE	N.A.	Hexa- through di-	N.A.			
	Sediments at the riverside of Lianjiang Rriver, Guiyu, E-recycling town in China		No	Nona- through tri-BDE	N.A.	N.A.	Too ambitious to correlate *Pseudomonas* with PBDE debromination simply by DGGE study in original microcosm	3 months	Qiu et al., 2012
	A few sediment slurries from mangrove, fresh water ponds, and marine subsurface sediments, Hongkong SAR		No	No	N.A.	Hexa 153 to hexa-, penta-, tetra-, tri-, and di-; tetra 47 to tri-	*Para*- removal preferred, followed by *meta*- and *ortho*-removal	90 days for tetra 47; 7.6 to 165 days for hexa 153	Zhu et al., 2014
	River sediment from Erren River, Taiwan (heavily contaminated rivers)		No	Nono-through mono-BDE	N.A.	N.A.		6 months	Huang et al., 2014
	Wastewater sludge samples, Hrade Kralove and Brno		No	A relative distribution of individual congeners changed with a significance increase of tetra 49			A mixture of mono-through hepta BDEs was spiked.	15 months	Stiborova et al., 2015
	E-waste contaminated soils		Lactate	Deca-209 and tetra 47 decrease while penta-99, hexa-154, 153, and hepta 183 increase at significant levels			PBDEs contamination already exist in soils samples; iron-reducing conditions		
	Mangrove sediment from Guandu and Bali, Taiwan		No	Nona-through di-	N.A.	N.A.	*Para*- removal preferred, followed by *meta*- and *ortho*-removal; Debrominating populations were tried to be identified through analysis of microbial communities; ZVI can enhance debromination	75 days	Yang et al., 2017
	Subsurface sediment from mature mangrove forest in Maipo, Hongkong SAR		No	N.A.	N.A.	Tetra- 47 to tri, di-	Biochar accelerates PBDEs' reductive debromination in electron transfer among microbial populations. Abundance of dehalogenating populations were enriched	20 weeks	Chen et al., 2018

Studies evaluating debromination potential with either technical PBDE mixtures or environmentally relevant BDE congeners in microcosms established from soils and sediments collected from various locations and environments have reported differences in both the rate and extent of PBDE debromination after long-term incubation (Table 2). When the primary goal is identification of functional bacteria, rather than developing enrichment cultures for fundamental studies, high-throughput sequencing and quantitative real-time PCR have been used to detect changes in microbial composition and identify specific populations whose increase correlates with debromination. Chen et al used this strategy to identify organohalide respiring *Dehalobacter, Dehalococcoides, Dehalogenimonas*, and *Desulfitobacterium* populations in a microcosm during reductive debromination of tetra-BDE 47 (Chen et al., 2018). Though some studies have purported to identify debrominating populations in microbial communities using less-sensitive molecular techniques [e.g., denaturing gradient gel electrophoresis (DGGE) and terminal restriction fragment length polymorphism (T-RFLP), Qiu et al., 2012; Huang et al., 2014], these results must be viewed skeptically. Organohalide respiring bacteria are typically minor populations, even within enrichment cultures, whose presence can be masked by more dominant non-debrominating populations, thus large changes in detected abundance that appear to be related to debromination often only represent small relative changes in abundance of the population—which is difficult to discern by DGGE or T-RFLP. In such cases, researchers may make erroneous conclusions about the identity of debrominating populations in the microbial community.

PBDEs debromination has been reported by bacteria of at least six different genera, and can be broadly divided as either metabolic process—energy from debromination to support cell growth, or co-metabolic process—not supporting cell growth (Tiehm and Schmidt, 2011). Co-metabolic dehalogenation typically requires supplementation with auxiliary substrates, e.g., other types of organohalides. Several studies of PBDEs debrominating microbial consortia and isolates have reported debromination only in the presence of additional halogenated electron acceptors (He et al., 2006; Robrock et al., 2008; Lee and He, 2010). Carbon sources may also act as auxiliary substrates, as in the case of the lactate, pyruvate or H_2_-CO_2_ dependent co-metabolic degradation of PBDEs identified in *Acetobacterium* sp. strain AG (Ding et al., 2013). The use of benign, non-halogenated auxiliary substrates in co-metabolic debromination makes strain AG a particularly interesting candidate for *in-situ* bioremediation. Co-metabolic reductive debromination of PBDEs is currently more frequently reported than metabolic debromination. However, metabolic reductive debromination is generally favored for site remediation because it does not require auxiliary substrates and has higher energy-utilization efficiency. Thus far, only *Dehalococcoides mccartyi* strains GY50 and GY52, both isolated from co-culture GY2 (Lee et al., 2011), have been shown to metabolically debrominate PBDEs (Ding et al., 2017). Strain GY50 is of particular interest as it completely debrominates penta-BDE mixtures to diphenyl ether, rather than producing partially debrominated end-products.

## Synergistic interactions in microbial reductive debromination of PBDEs

Synergistic metabolic interactions between organohalide respiring bacteria and other bacterial populations can increase the robustness of dehalogenation in microbial consortia by providing certain growth factors (He et al., 2007; Men et al., 2011). Although the exact nature of most of these synergisms is unknown, faster and extensive debromination in mixed microbial communities has been observed. Co-metabolic debromination in co-culture G consisting of *D. mccartyi* strain DG and *Acetobacterium* sp. strain AG, with presence of auxiliary substrate, TCE, is an example of this synergistic metabolic interactions (Ding et al., 2013). Culture G, a co-culture originating from a river bank, was found to reductively debrominate octa- and penta-BDE mixtures to less brominated congeners ranging from penta- to di-BDEs. Debromination of the BDE mixtures by strain DG was slower and also less extensive than that in the parent culture, by producing only trace amounts of penta- and tetra-BDEs after 6 weeks' incubation. Strain AG had the same penta-BDEs debromination capacity as culture G, albeit more slowly and less extensively than its parent culture, and had an octa-BDE debrominating profile similar to that of strain DG. Debromination of octa-BDE could be rescued in co-cultures of strain AG and strain DG, indicating a synergistic relationship between these two populations. The authors speculated that the *Acetobacterium* provides certain growth factors, such as vitamin B_12_ in the form of cobalamin, which are essential for PBDEs debromination by strain DG.

Besides supplying essential nutrients during synergistic interactions, another benefit of synergetic microbial interactions can be seen in the more extensive debromination of PBDEs by culture EC195 plus strain BAV1 compared to culture EC195 alone, as direct participants in debromination processes (He et al., 2006). The highly enriched autotrophic culture EC195 produced hepta- through di-BDEs from an octa-BDE mixture and *D. mccartyi* strain BAV1 did not debrominate octa-BDEs in pure culture. However, addition of strain BAV1 to EC195 resulted in further debromination, with generation of only tetra- through di-BDEs from the octa-BDE mixture. The hexa- and penta-BDE debromination by EC195 plus strain BAV1 suggests a role for BAV1 in synergistic debromination of lesser brominated PBDEs.

However, interactions among populations in mixed microbial communities do not necessarily improve debromination activity. For example, inhibition of methanogenic bacteria in culture GY-T-2 by the addition of BES increased the rate and extent of the debromination of PBDEs (Lee and He, 2010). It is possible that methanogens in GY-T-2 compete with the PBDE debrominating populations in the community for limiting factors, such as hydrogen, thereby inhibiting debromination.

## Reductive debromination of PBDEs and functional genes

Anaerobic reductive debromination of PBDEs was first observed in a bioreactor where di-BDE 15 was converted to mono-BDE 3 and diphenyl ether (Rayne et al., 2003). Later investigations of biological transformation of PBDE mixtures and some individual congeners (e.g., tetra-BDE 47, penta-BDE 99) identified and characterized anaerobic microbial reductive debromination of PBDEs in sewage sludge treatments as well as in terrestrial, marine, and estuarial soils and sediments, and in microbial consortia and isolates derived from these sources (Gerecke et al., 2005; He et al., 2006; Robrock et al., 2008; Tokarz et al., 2008; Lee and He, 2010; Lee et al., 2011; Xu et al., 2014; Zhu et al., 2014; Stiborova et al., 2015).

Debromination of PBDEs is partial in almost all observed systems, yielding lesser brominated metabolites, and typically occurs slowly and at nanomolar concentrations. Gerecke et al. reported microbial debromination of 5% of 11.2 nM deca-BDE 209 to nona- and octa-BDEs after 238 days in a microcosm established from sewage sludge (Gerecke et al., 2005). A separate study utilizing a *D. mccartyi*-containing microbial consortia, ANAS195, observed production of 500 nM hepta- to di-BDEs from 1.3 μM of an octa-BDE mixture with the presence of TCE after 6 months' incubation (He et al., 2006). Similarly, partial debromination of deca-BDE 209 and tetra-BDE 47 was reported after 90 days incubation by the autochthonous microbial community in e-waste contaminated soil containing a range of deca- to tri-BDEs (Song et al., 2015). Though uncommon, complete debromination of tetra- and penta-BDEs to diphenyl ether was demonstrated by Lee et al in a co-culture, GY2 (Lee et al., 2011), from which a novel *D. mccartyi* strain, GY50, that debrominated ~1,180 nM tetra-BDE 47, penta-BDEs 99 and 100 to diphenyl ether in 2 weeks was isolated (Ding et al., 2017).

There is a marked variation in observed debromination rates, pathways, and relative abundance of daughter products among different studies of anaerobic microbial debromination. This is most likely due to regional differences in nutrient availability and bioavailability of PBDEs as well as site-specific variations in microbial community composition. However, it is generally true that more highly substituted PBDEs (deca- and octa-BDEs) are debrominated by fewer organisms and more slowly than lesser substituted congeners (penta- and tetra-BDEs), which is a common trend in microbial dehalogenation of aromatic organohalides [i.e., PBDEs, polychlorinated biphenyl ethers (PCBs), polychlorinated dibenzo-p-dioxins and polychlorinated dibenzofurans (PCDD/Fs), etc.]. This is thought to be a result of the increased hydrophobicity of more highly halogenated aromatics and of the reduced reactivity of more highly substituted aromatic rings resulting from changes in electron density (Fagervold et al., 2007; Cooper et al., 2015; Zhang et al., 2017, 2018). Instances of this phenomenon can be seen in several studies in which prevalence of octa-BDEs debromination was observed in pure cultures and defined mixed cultures than debromination of deca-BDEs (He et al., 2006; Robrock et al., 2008; Xu et al., 2014). Similarly, studies of the debromination potential of municipal sewage sludge (Shin et al., 2010) and various sediment slurries (Zhu et al., 2014) have noted debromination of hexa- to tetra-BDEs but no debromination of deca- or hepta-BDEs.

**Figure 3:**
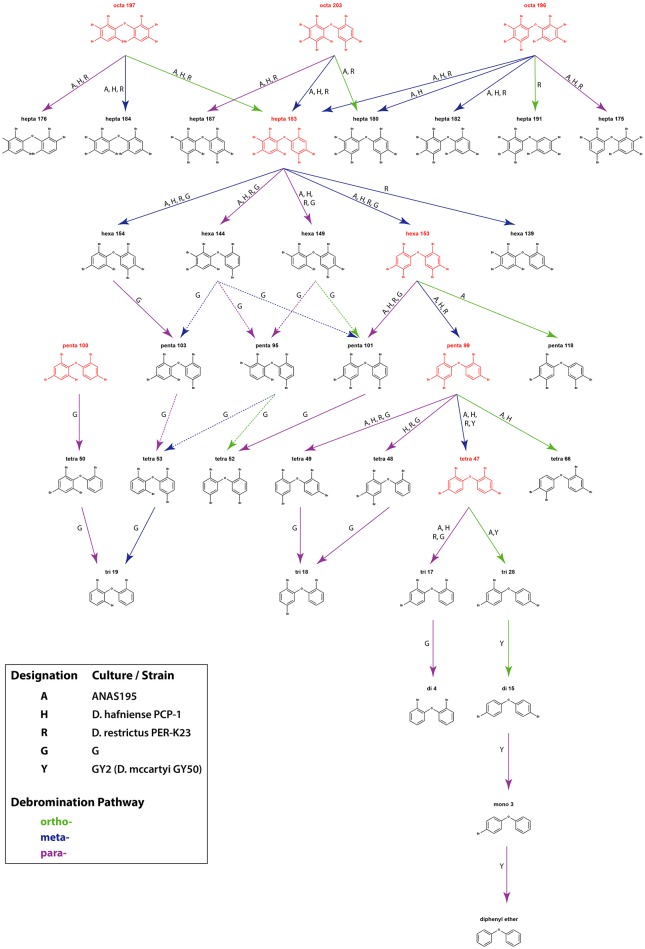
Debromination pathways of PBDEs in mixed and pure microbial cultures.

Attempts to identify the products of anaerobic microbial debromination have also revealed preferential removal of *para* and *meta* bromine substituents (Figure 3), a trend which is also present in microbial dehalogenation of aromatic organohalides, such as PCBs (Wang and He, 2013; Wang et al., 2014). This preference appears to exist regardless of the degree of bromine saturation, and has been observed in debromination of deca-BDE 209 (Gerecke et al., 2005) as well as in debromination of octa-BDEs (BDE 196, 203, and 197), hepta-BDE 183, hexa-BDE 153, penta-BDE 99, and tetra-BDE 47 (Robrock et al., 2008). The 2008 study by Robrock et al. also suggests preferential removal of double-flanked bromine moieties. Strict *para* and *para*-dominant debromination patterns in penta- and octa-BDE mixtures are also found in culture G with the presence of TCE, although *meta-* and *ortho-*bromine substitution were suggested as minor pathways in octa-BDE debromination (Ding et al., 2013). The only exception thus far identified not following preferential *para* and *meta* substitution is the predominance of *ortho*-bromine removal in co-culture GY2, as well as in *D. mccartyi* strain GY50 (derived from culture GY2), in stepwise conversion of penta-BDE 100 to di-BDE 15 via tetra-BDE 47 and tri-BDE 28 (Lee et al., 2011).

The preference on *para-, meta-*, or *ortho*-bromine substitution could be determined by the reductive dehalogenases present in the debrominating bacteria. Identification and characterization of reductive dehalogenases responsible for dehalogenation of specific compounds allow researchers to investigate the mechanisms of organohalide respiration and provide targets that can be used to monitor populations of organohalide respiring bacteria in laboratory and field-scale bioremediation studies. Reductive dehalogenases responsible for dehalogenation of a wide variety of halogenated compounds have been reviewed in Hug et al. (2013). Identification of PBDEs reductive dehalogenases is impeded by the co-metabolic nature of PBDEs debromination in most mixed cultures and isolates, as the presence of auxiliary substrates makes it more challenging to determine single gene products responsible for observed activities, and by marginal cell yield of PBDEs debrominating populations in mixed cultures.

**Figure 4:**
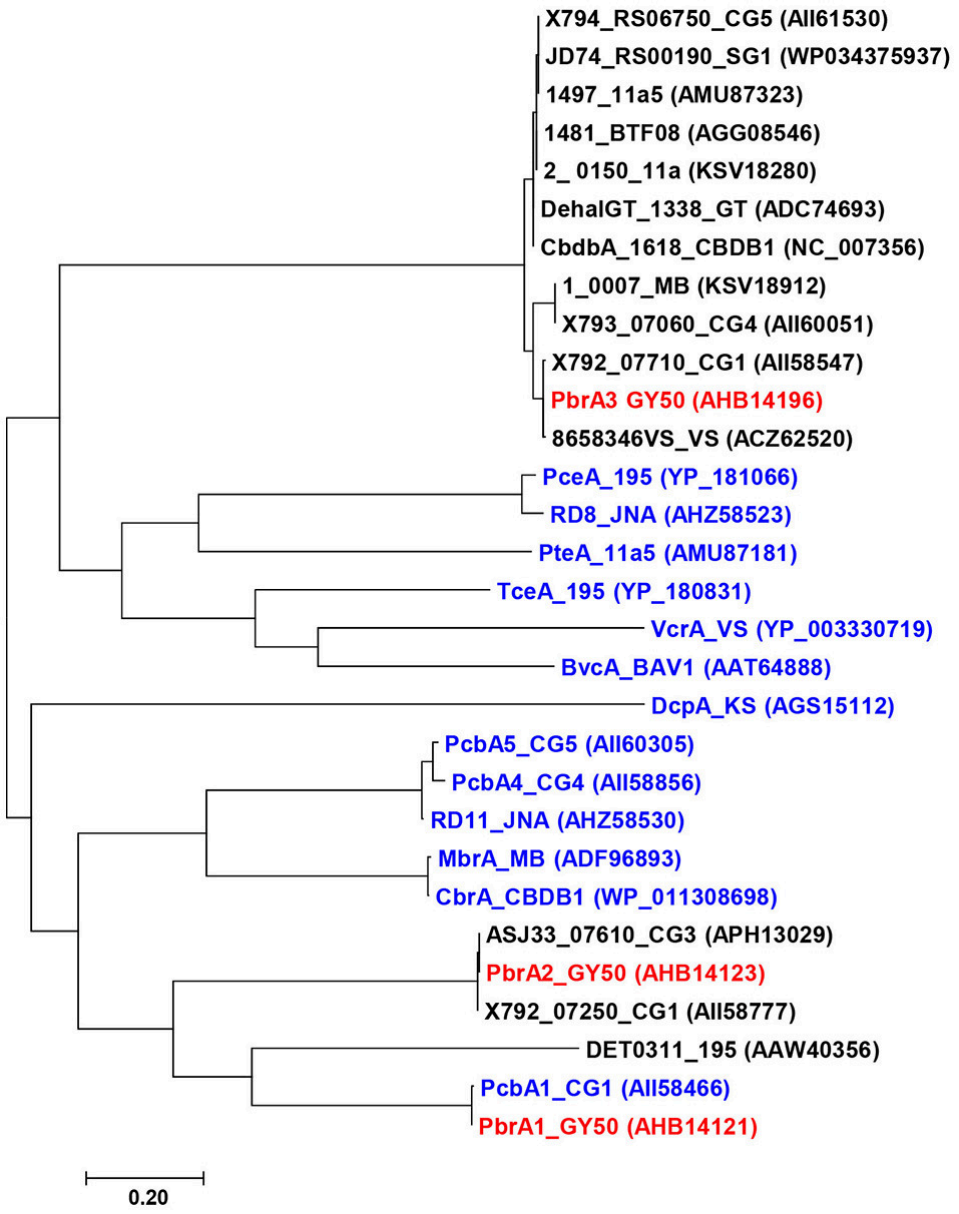
Phylogenetic analysis of functionally characterized RDases (blue color) in *Dehalococcoides mccartyi* including orthologs (black color) of PBDE reductive dehalogenases (red color). The tree was constructed with MEGA 7 (Kumar et al., 2016) using maximum likelihood method in JTT matrix-based model (Jones et al., 1992).

The only PBDE reductive dehalogenases characterized to date, PbrA1, PbrA2, and PbrA3, were identified in *D. mccartyi* strain GY50 using a combination of transcriptomics and proteomics (Lee et al., 2011; Ding et al., 2017). The fortuitous emergence of two variant strains that exhibited distinct dehalogenation profiles to strain GY50 allowed for functional characterization of the three PBDE dehalogenases. The deletion of a genomic island containing both PbrA1 and PbrA2 in the genome of strain GY52 and the lack of di-BDE debromination in this strain provided strong evidence for the role of PbrA3 in debromination of penta- and tetra-BDEs to di-BDE (BDE 15), while simultaneously implicating PbrA1 and PbrA2 in removal of unflanked para-bromines from BDE 15 to mono-BDE (BDE 4) and diphenyl ether. Several hundred putative reductive dehalogenase homologous genes have been identified in the genomes of different *Dehalococcoides* strains and have been categorized into more than 50 orthologous groups based on amino acid similarity (Hug et al., 2013). The three PBDE reductive dehalogenases, PbrA1, PbrA2, and PbrA3, which catalyze different debromination pathways and attack bromine moieties at different positions, are phylogenetically distinct—sharing < 40% amino acid sequence similarity with each other (Figure 4). It is not uncommon for reductive dehalogenase genes with similar functionality to exhibit significant disparities in nucleotide sequence, but the phylogenetic similarity of the PBDE reductive dehalogenases to enzymes which catalyze dehalogenation of other poly-halogenated aromatic compounds may reveal some structural aspect that is common among enzymes mediating catalysis of these types of organohalides. While neither nucleotide nor amino acid sequence similarity among reductive dehalogenases is predictive of substrate range (Hug, 2016), the clustering of known reductive dehalogenase into orthologous groups at least provides a starting point for putatively identifying other PBDE reductive dehalogenases. For example, debromination of PBDEs and other brominated benzenes (Wagner et al., 2012) and phenols (Yang et al., 2015) by strain CBDB1 contains one reductive dehalogenase gene that clusters within the same ortholog group as PbrA3, though no functional genes were implicated in the original study. More information about the functions of uncharacterized dehalogenases is necessary before meaningful comparative analyses of these orthologs can be performed. The emergence and characterization of the GY52 variant facilitated description of the regiospecificity of these three dehalogenases and demonstrated a strategy by which other PBDE dehalogenases can be investigated.

## Outlook

Though production and usage of PBDE mixtures have declined after implementation of bans and voluntarily cessation of manufacture, the environmental persistence and potential for transboundary disbursement make PBDEs a continuing threat to biological populations around the globe. The degree of the debromination of PBDEs not only affects physical and chemical properties, but also toxicity and potential for bioaccumulation. Highly substituted deca-, nona-, and octa-BDEs are thermally labile and are partially degraded to lesser brominated congeners in the environment resulting in an increase in the risk presented by the original contamination. Because PBDEs preferentially sorb to organic matter, they tend to accumulate in anaerobic and anoxic soils and sediments. Harnessing the metabolic potential of anaerobic microbes that can detoxify PBDEs by removing bromine substituents has the potential to be a cost effective and efficient approach to remediate PBDEs in the environment.

Microbial debromination of PBDEs must overcome several obstacles before it can be considered a viable technology for bioremediation. Most of the bacteria that are currently known to debrominate PBDEs do so co-metabolically and partially. This incomplete debromination may often cause additional problems *in-situ* and the requirement for auxiliary substrates can severely limit the rate and extent of debromination. The only anaerobic microbe that can completely detoxify PBDEs and couple cell growth is *D. mccartyi* GY50 which can metabolize penta- and tetra-BDEs to produce diphenyl ether as an end-product. However, since deca- and octa-BDE mixtures also represent the majority of PBDEs production and pollution globally, further study is necessary to find other bacterial isolates and mixed cultures that can metabolize these highly brominated congeners. In general, co-cultures could likely be a promising solution to completely debrominate higher brominated BDEs to diphenyl ether via intermediates such as penta- and tetra-BDEs.

The slow growth rate and low cell yields associated with debrominating bacteria have impeded efforts to elucidate the mechanisms of microbial PBDE degradation. The isolation of *D. mccartyi* GY50 has revealed functional PBDE reductive dehalogenases for the first time, which may facilitate identification of additional PBDE dehalogenases in other *Dehalococcoides*. Discovery of enzymes responsible for the observed debromination of higher brominated deca- and octa-BDEs would be particularly valuable to the advancement of PBDEs bioremediation efforts. Recent advances in heterologous expression and purification of functional reductive dehalogenases will facilitate investigations into the dehalogenation potential of uncharacterized dehalogenases and may make it possible to establish a platform for *in-vitro* production of specific dehalogenases for bioremediation.

In summary, the innate capacity of some anaerobic microbes to detoxify different PBDEs can potentially be exploited as a tool to remediate contaminated soils and sediments. Application of these microbes *in-situ* has been hindered by the slow rate of cell growth and associated debromination of target compounds. Combining organohalide respiring bacteria with other physical and chemical processes to increase the rate and extent of anaerobic debromination of PBDEs has been investigated with varying degrees of success. Recent descriptions of PBDE debrominating isolates and defined microbial consortia have shed light on genes responsible for some, but not all, of the natural attenuation of PBDEs that has been observed. Future investigations to elucidate and characterize additional PBDEs dehalogenases in anaerobic systems may provide a clearer picture of the mechanisms responsible for the partial degradation of highly substituted PBDEs in the environment and pave the way for development of new strategies to address the persistent threat that PBDEs pose to biological populations.

## Ethics statement

This article does not contain any studies with human participants or animals performed by any of the authors.

## Author contributions

SZ, MR, and JH developed the structure of the article. SZ and MR drafted the article. SZ, MR, CD, and JH revised the article. JH did the final approval of the version to be published.

### Conflict of interest statement

The authors declare that the research was conducted in the absence of any commercial or financial relationships that could be construed as a potential conflict of interest.
